# Exosomes based advancements for application in medical aesthetics

**DOI:** 10.3389/fbioe.2022.1083640

**Published:** 2022-12-20

**Authors:** Bin Zhang, Jianmin Gong, Lei He, Adeel Khan, Tao Xiong, Han Shen, Zhiyang Li

**Affiliations:** ^1^ College of Life Science, Yangtze University, Jingzhou, China; ^2^ Department of Clinical Laboratory, The Affiliated Drum Tower Hospital of Nanjing University Medical School, Nanjing, China; ^3^ State Key Laboratory of Bioelectronics, School of Biological Science and Medical Engineering, National Demonstration Center for Experimental Biomedical Engineering Education, Southeast University, Nanjing, China

**Keywords:** exosomes, engineering production, preserve, separate, medical aesthetics

## Abstract

Beauty is an eternal pursuit of all people. Wound repair, anti-aging, inhibiting hyperpigmentation and hair loss are the main demands for medical aesthetics. At present, the repair and remodeling of human body shape and function in medical aesthetics are often achieved by injection of antioxidants, hyaluronic acid and botulinum toxin, stem cell therapy. However, there are some challenges, such as difficulty controlling the injection dose, abnormal local contour, increased foreign body sensation, and the risk of tumor occurrence and deformity induced by stem cell therapy. Exosomes are tiny vesicles secreted by cells, which are rich in proteins, nucleic acids and other bioactive molecules. They have the characteristics of low immunogenicity and strong tissue penetration, making them ideal for applications in medical aesthetics. However, their low yield, strong heterogeneity, and long-term preservation still hinder their application in medical aesthetics. In this review, we summarize the mechanism of action, administration methods, engineered production and preservation technologies for exosomes in medical aesthetics in recent years to further promote their research and industrialization in the field of medical aesthetics.

## 1 Introduction

Beauty is the eternal pursuit of all people. Wound repair (Prasai et al., 2022), anti-aging (Hu et al., 2019), inhibiting hyperpigmentation (Wang et al., 2021) and inhibiting hair loss (Yang G. et al., 2019) are the main demands for medical aesthetics. Medical aesthetics is a perfect combination of regenerative medicine and improvement of one’s looks (Edmonds, 2013). It repairs, replaces, or regenerates human cells and tissues by using regenerative medicine technologies such as cells, natural or artificial scaffold materials and growth factors (Lee J. et al., 2020; Huang et al., 2021). At the same time, it is used in medical aesthetics to achieve the repair, remodeling and improvement of human appearance, shape and function, so as to achieve the harmony and improvement of aesthetics, medicine, shape and function of the human body (Martínez-González et al., 2019; Xiong et al., 2021).

At present, in the field of medical aesthetics, anti-oxidants such as vitamin C (Odrobinska et al., 2020) and resveratrol (Ratz-Lyko and Arct, 2019), hyaluronic acid (Shekhter et al., 2019; Gazitaeva et al., 2021) and botulinum toxin (Naik, 2021) are often injected to remove wrinkles, reduce color spots and promote wound healing. However, there are still many challenges in aesthetics, for example, it is difficult to control the dose of anti-oxidant injection (Shekhter et al., 2019; Gazitaeva et al., 2021). In clinical practice, fat transplantation is often used in medical aesthetics, but it may lead abnormal local contour shape and to increase foreign body sensation (Yan et al., 2021). Subsequently, stem cell therapy was gradually applied to regenerative medicine due to its pluripotency, self-renewal and ability to promote the secretion of regenerative cytokines (Johari et al., 2019; Li W. et al., 2022), but stem cell therapy may induce the risk of tumorigenesis and malformation (Trounson and McDonald, 2015). Moreover, exosomes are nano vesicles secreted by cells, which belong to one kind of extracellular vesicles and play an important role in intercellular communication (Mehryab et al., 2020). Compared with drugs, plant/herbal extracts and bioactive molecules, exosomes have the advantages of low immunogenicity, good biocompatibility, targeting specificity and strong tissue permeability (Ha et al., 2016; Liang et al., 2021; Rao et al., 2021). They are often used as drug delivery vehicles and can play a role in medical aesthetics (Li M. et al., 2020). Here, we summarize the mechanism of action, administration methods, engineered production, separation, purification and presser-vation methods of exosomes in the field of medical aesthetics in recent years with a view to furthering the research and industrialization process of exosomes in the field of medical aesthetics.

## 2 Biological properties of exosomes

Exosomes have unique physicochemical properties, such as their ability to pass through tissue barriers, mononuclear phagocytic cell systems and certain targeting properties (Marcus and Leonard, 2013) when carrying drugs, and they are often used as therapeutic drug delivery carriers in medical aesthetics. For example, exosomes’ lipid bilayer structure can prolong drug circulation time *in vivo*, escaping the elimination by mononuclear phagocytosis system, increasing the local drug concentration, and effectively controlling the drug release (Nam et al., 2020). Compared with traditional nanomaterials, exosomes have good biocompatibility, degradability, low toxicity, and low immunogenicity, so they are more suitable as drug delivery carriers (O'Brien et al., 2020).

### 2.1 Structure and composition of exosomes

**FIGURE 1:**
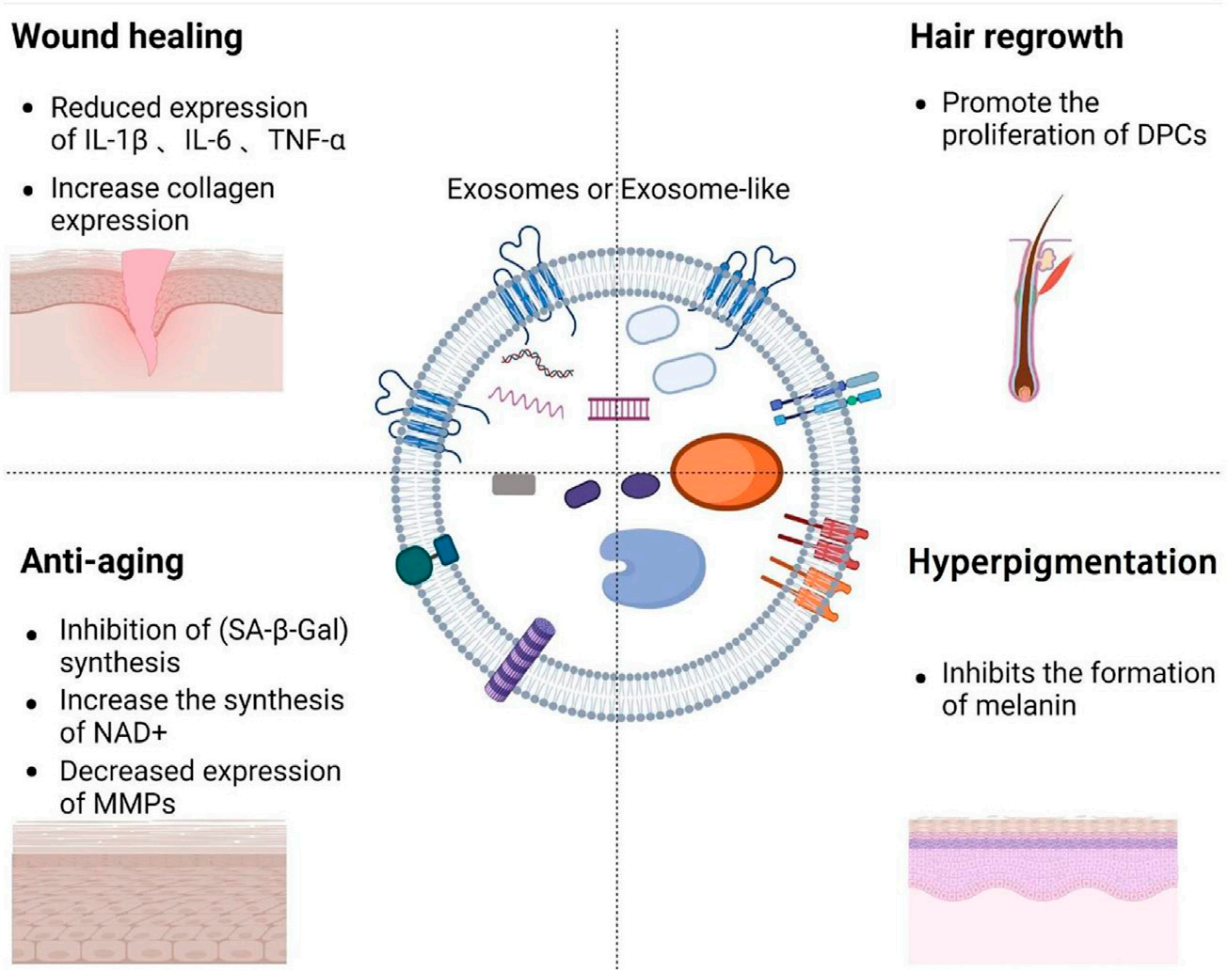
Overview of the composition of exosomes and the role of exosomes in medical aesthetics.

The lipid bilayer membrane structure of exosomes protects the abundant proteins, nucleic acids, microRNAs (miRNAs), cholesterol and sphingomyelin in the membrane from being degraded (Pegtel and Gould, 2019; Li X. et al., 2020; Foo et al., 2021; Gurunathan et al., 2021) (Figure 1). A common cytoplasmic protein in exosomes is the RAB protein, a member of the guanylate triphosphatase (GTPases) family, which regulates the fusion of exosome membranes with recipient cells (An et al., 2021). In addition to RAB proteins, exosomes are rich in annexins that have exosomal membrane exchange and fusion effects (Tan et al., 2017; Keklikoglou et al., 2019). Exosomes membrane is rich in tetraspanins involved in exosomes transport family (CD63, CD81, and CD9) (Barranco et al., 2019) and heat shock protein family (HSP60, HSP70, and HSP90) (Regimbeau et al., 2021). Exosomes transport cargoes through the lipid bilayer membrane, and can deliver active ingredients (including proteins, nucleic acids, and lipids) from parent cells to recipient cells (Villarroya-Beltri et al., 2014; van Niel et al., 2018), and they can selectively enter target cells (Li M. et al., 2020) by homing to target tissues. Their active ingredients are delivered to the target cells’ cytoplasm, thereby changing recipient cells’ physiological state (Tkach and Thery, 2016).

### 2.2 Biogenesis

In the aspect of medical aesthetics, the research of exosomes biogenesis is crucial to the engineering transformation of exosomes and the production of larger quantities. A short overview of exosomes biogenesis can be stated as the inward movement of cytoplasmic membranes results in wrapping around extracellular entities and various membrane proteins forming the early sorting endosomes (ESEs), the ESEs fuses with other ESEs to form late sorting exosomes (LSEs). The LSEs develop into multivesicular bodies (MVBs). MVBs contain many intraluminal vesicles (ILVs) that are released into exosomes. After its formation, the MVBs can either be degraded by fusion with lysosomes or fuse with the plasma membrane with ILVs in it, the final exosomes (Borges et al., 2013; Matic et al., 2020; Ras-Carmona et al., 2021).

**FIGURE 2:**
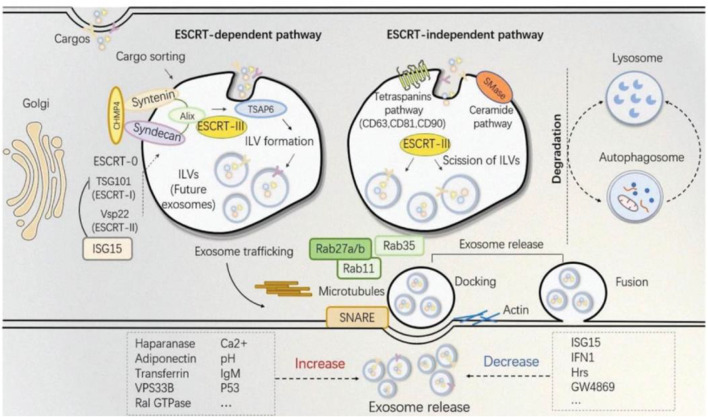
The established theory of exosomes biogenesis indicates different pathways distinguished as ESCRT-dependent and ESCRT-independent (Shi et al., 2021).

Because the biogenesis of exosomes is mainly divided into two ways: dependent on the transport of necessary endosome sorting complex (ESCRT) pathway and independent of ESCRT. Therefore, we herein discuss some ESCRT-dependent and ESCRT-independent mechanisms to increase exosome production (Figure 2). These following methods provide important ideas on engineering preparation of related exosomes in the field of later medical aesthetics and improve their production rate. ESCRT-dependent pathways: Genetic manipulation of gene generation pathways to overexpress activating genes for exosome biogenesis and downregulating key regulatory genes involved in exosome transport, storage, secretion, or recycling. In most of these pathways, genes have a directly positive effect on exosome production (Jafari et al., 2020). For example, Wang et al. found that overexpression of HSP20 attenuated diabetes-induced cardiac damage. In addition, the elevation of HSP20 promoted the secretion of exosomes by directly interacting with Tsg101, a promoter of the exosome biogenesis pathway, and the production of exosomes was increased by 1.8-fold compared with the control group (Wang et al., 2016) (Table 1 for details).

**TABLE 1:** Take parts as an example: Gene manipulation promotes exosome release.

Modification	Cell line	Outcome	Mechanism of action	References
Overexpression of HSP20	Cardiomyocyte	Increased generation of exosomes	Direct interaction of Hsp20 with Tsg101	Wang et al. (2016)
Overexpression of LMP1	HEK293	Separation EV increased 2–4 times	CD63-mediated exosomes LMP1 release	Hurwitz et al. (2017)
Overexpression of TSPAN6	HEK293	Exosome release increased by 60%	Interaction with syntenic	Guix et al. (2017)
Cortactin overexpression	SCC61	Exosome secretion increased 1.5–2 times	Interaction with Arp2/3 complex and F-actin	Sinha et al. (2016)
Overexpression of the tetraspanin CD9	Standard HEK-AAV producer	The production of exosomes increased 3.75 times	Not mentioned	Schiller et al. (2018)
Combined expression of STEAP3, syndecan-4	HEK293	The production of exosomes increased 15–40 times	Not mentioned	Kojima et al. (2018)
miR-126-3p-overexpressing combined with chitosan wound dressing	SMSCs	Increased exosome release	Not mentioned	Tao et al. (2017)

ESCRT independent pathway involves steps such as cell culture manipulation *via* altering the media, use of specific drugs and harsh conditions to accelerate the production of exosomes. For example, Ban et al. isolated exosomes from cell culture media at pH 4, 7 and 11, and found that the concentration of exosomes protein and RNA in pH 4 medium was 5 times higher than that in pH 7 medium (Ban et al., 2015). However, one study showed that storage at pH 4 would reduce the concentration of exosomes and increased the absorption of exosomes by cells (Cheng et al., 2019), but at present many reports still use pH 7 buffer to preserve exosomes (Salimu et al., 2017; Petersen et al., 2018). Therefore, the influence of pH value on exosomes still needs to be further explored in the future. Together, we can broaden, change or improve the therapeutic capacity of exosomes by studying or engineering their structure, biogenesis, and properties (Table 2 for detailed data).

**TABLE 2:** Physical or chemical methods promote the release of exosomes.

Condition	Cell line	Outcome	Mechanism of action	References
Acidic pH	HEK293	Production of exosomes increased 5 times	Acidic pH could increase the stability of exosomes	Ban et al. (2015)
Oxidative stress	ARPE-19	The release of the exosomes containing the VEGR receptor was increased 4 times	RPE cells release higher amounts of exosomes when they are under oxidative stress	Atienzar-Aroca et al. (2016)
Glucose starvation	H9C2	Increased exosome release	Not mentioned	Garcia et al. (2015)
Hypoxia	MCF7, SKBR3, and MDA-MB-231	Exosome release increases 1.4-2 times	Activation of hypoxic signaling by dimethyloxalylglycine, elevation of miR-210	King et al. (2012)
Incubation with IgM	CLL	Secretion of exosomes increases by about 2 times, expression of miR-150 and miR-155 in exosomes	B cell receptor activation by a-immunoglobulin (Ig)M induces exosome secretion	Yeh et al. (2015)
Cyclophosphamide	LBC T cell	Exosomes increased by 61%	Not mentioned	Cocozza et al. (2019)
Recombinant WNT5A	Melanoma cells	Increased exosome release	Ca^2+^-dependent release of exosomes containing IL-6, VEGF and MMP2 proteins	Ekström et al. (2014)
Extracellular Ca^2+^	SJSA-1, Hs578T	The production of tumor microvesicles and the formation of tumor globules increased about 3 times	Not mentioned	Crawford et al. (2010)

## 3 The regulatory mechanism of exosomes in medical aesthetics

At present, several studies have proved that miRNAs (Zhai et al., 2020), related active factors (Yoshida et al., 2019) in exosomes mediate wound repair (Li and Wu, 2022), anti-aging (Han et al., 2022), inhibiting hyperpigmentation (Liu et al., 2019), and inhibiting hair loss (Bae and Kim, 2021) and other related signaling pathways, regulating inflammatory factors interleukin-1β (IL-1β) (Li Z. Q. et al., 2020), matrix metalloproteinase 1 (MMP-1) (Liu et al., 2021), matrix metalloproteinase 3 (MMP-3) (Wang et al., 2017), collagen 1 (COL1A) and collagen 3 (COL3A) (Qi et al., 2020) expression of related genes, thus playing a role in medical aesthetics. For example, mesenchymal stem cell exosomes (MSCs-EXOs) encapsulated miR-223 regulate the polarization of macrophage M2 by targeting pknox1 and reducing the related inflammatory factor IL-10,TNF-α expression, thereby controlling the inflammatory response (He et al., 2019). Below we specifically explore exosomes’ role and regulatory mechanism in wound repair, anti-aging, inhibiting hyperpigmentation, and inhibiting hair loss (Table 3 for details).

**TABLE 3:** Role of exosomes in medical aesthetics.

Source	Mechanism of action	Function	References
Mesenchymal stem cell exosomes	miR-223 coated by MSCS-Exos regulates M2 polarization of macrophages by targeting Pknox1	Wound healing	He et al. (2019)
Keratinocyte-derived exosomes	Carrying miR-330-5p inhibits melanin production by targeting TYR	Hyperpigmentation	Liu et al. (2019)
Exosomes derived from human amniotic stem cells	miR-181a-5p and miR-199a, respectively, inhibit melanin production by reducing MITF expression	Hyperpigmentation	Wang et al. (2021)
Milk exosomes	miR-2478 directly targets rap1a *via* the Akt-GSK3 β pathway as a regulator of Melanin production, which reduces Melanin content in melanocytes and inhibits Melanin formation	Hyperpigmentation	Bae and Kim, (2021), Han et al. (2022)
Fat Mesenchymal stem cell exosomes	By regulating miR-22, Wnt/β-catenin signal pathway and TNF-α signal pathway, the proliferation and migration of DPCs and expression of ALP, versican and Alpha-smooth muscle actin (α-SMA) proteins were promoted	Control hair loss	Nilforoushzadeh et al. (2020), Li et al. (2022b)
Human-induced potent stem cell-derived exosomes	It decreased the activity of SA-β-Gal and inhibited the expression of P53 and P21 in HDFs	Anti-aging	Lee et al. (2020a)
Blood exosomes	NAMPT carried in exosomes increases the biosynthesis of NAD	Anti-aging	Yoshida et al. (2019)
Endothelial progenitor cells exosomes	Activation of ERK1/2 signal pathway enhances the ability of human endothelium to proliferate, migrate and become tube	Wound healing	Zhang et al. (2016b)
Exosomes derived from human umbilical Mesenchymal stem cell	Activation of ERK pathway significantly inhibits Melanin Synthesis during MITF degradation	Hyperpigmentation	Kim et al. (2015)
Exosomes derived from Dermal Papilla cells	Down-regulation of relevant hair follicle inhibitory signal proteins by genes involved in the key pathways of β-catenin, WNT, BMP2 and BMP4 promotes the proliferation of hair follicle stem cells	Control hair loss	Zhou et al. (2018), Zhang et al. (2022)
Exosomes derived from Human Mesenchymal stem cell	Activation of hair inductivity of DPCs, AKT phosphorylation, Bcl-2 in Dermal Papilla, and regulation of proliferation of DPCs	Control hair loss	Rajendran et al. (2017), Taghiabadi et al. (2020)
Fat Mesenchymal stem cell exosomes	Inhibit the over-expression of MMP-1, MMP-2, MMP-3 and MMP-9 induced by UV irradiation, and enhance the expression of Collagen type I and III and Elastin	Anti-aging	Choi et al. (2019)
Wheat exosomes	The gene expression related to wound healing was enhanced and gene modification coordinated the formation of blood vessel	Wound healing	Sahin et al. (2019)

### 3.1 The role and mechanism of exosomes in wound repair

Skin wound repair is a complex dynamic physiological process (Jackson et al., 2012; Ogawa, 2017). Studies have shown that exosomes play a role in promoting blood coagulation (Zifkos et al., 2021), reducing inflammation (Chamberlain et al., 2021), accelerating tissue remodeling (Das et al., 2019), and inhibiting scar formation (An et al., 2021) by mediating wound repair-related signaling pathways such as MAPK (Gartz et al., 2018) and ERK (Geng et al., 2021). Exosomes can be vital in wound repair.

#### 3.1.1 Procoagulant

At present, the mechanism and physiological relevance of exosomes for procoagulant effect in wound repair is still unclear. However, exosomes are rich in active components and have unique physicochemical properties, such as passing through tissue barriers, which are beneficial in reducing blood coagulation in human blood. Studies have found that salivary exosomes can activate TF and coagulation factor VII-mediated coagulation in human plasma, helping coagulation, thereby reducing the risk of blood loss and pathogens entering the blood, thus contributing to innate immunity and host defense (Berckmans et al., 2011).

#### 3.1.2 Reduction of inflammation

**FIGURE 3:**
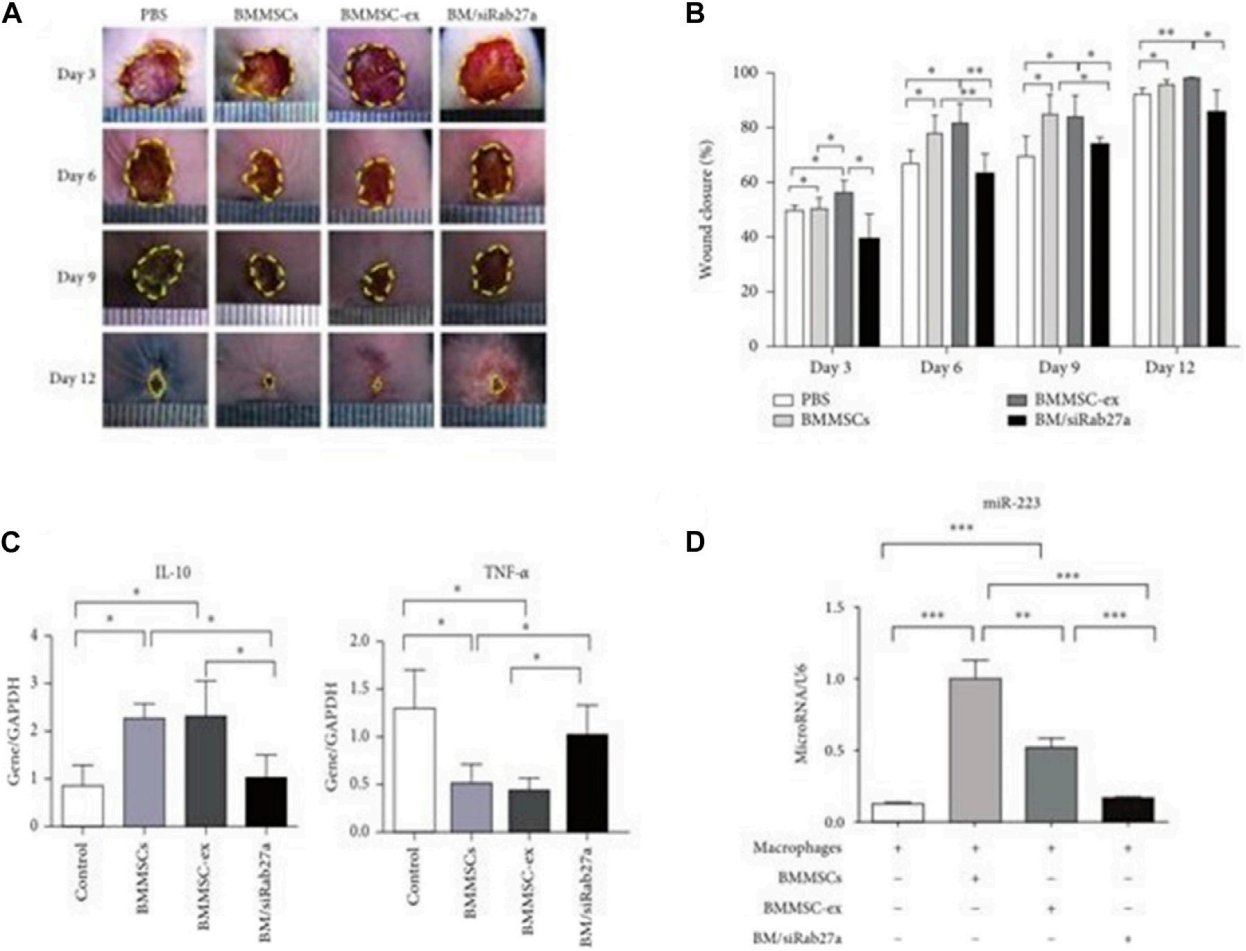
Utlization of MSCs derived exosomes for wound healing. **(A)** Depicts the light field photographs of cutaneous wounds post treatment with PBS, BMMSCs, BMMSC-ex, and BM/siRab27a. **(B)** Percentage of the wound closure on day 3–day 12 in reference to the day 0 wounds (*n* = 4). **(C)** qRT-PCR analysis of IL-10 and TNF-α in macrophages after being cocultured with BMMSC-, BMMSC/siRab27a-, or BMMSC-derived exosomes (*n* = 3). **(D)** qRT-PCR analysis of miR-223 in macrophages cocultured with BMMSCs, BMMSC-ex, and BM/siRab27a (He et al., 2019).

Skin damage causes an inflammatory response, and there are also changes in the secretion of inflammatory factors (Marofi et al., 2021). Exosomes can reduce the time from wound repair inflammation to remodeling by reducing the expression of related inflammatory factors. Studies have shown that mesenchymal stem cell exosomes (MSCs-EXOs) encapsulated miR-223 regulate the macrophage M2 polarization by targeting pknox1 and reduces the related inflammatory factor IL-10, NF-α expression, thereby controlling the inflammatory response (He et al., 2019) (Figure 3). These results indicate that exosomes can accelerate the transition of wound repair from the inflammatory phase to the remodeling phase, thereby promoting wound repair.

#### 3.1.3 Accelerated tissue remodeling

**FIGURE 4:**
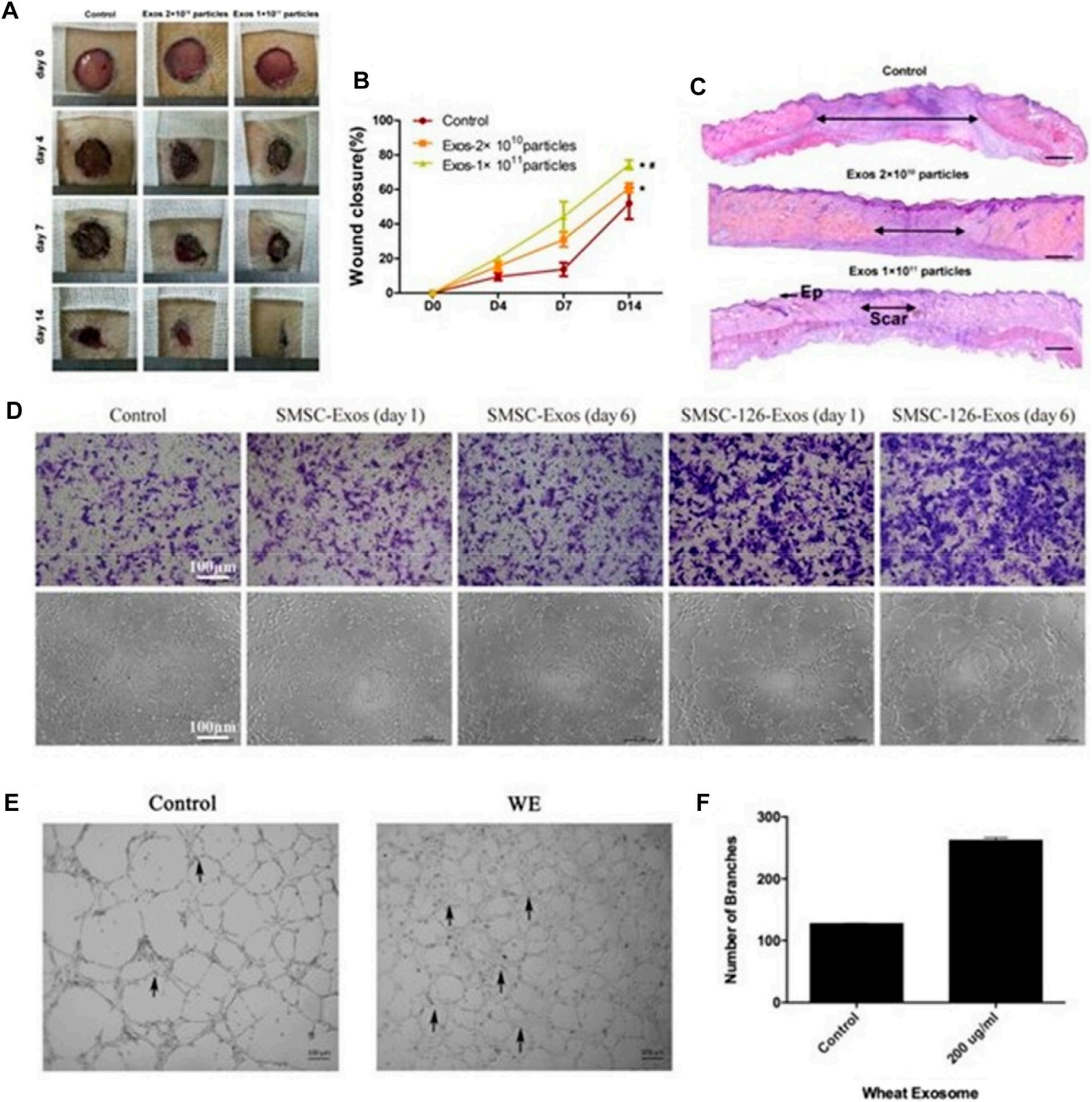
Exosomes can accelerate tissue remodeling. **(A)** General view of wounds treated with PBS or EPC exosomes of different concentrations on the 4th, 7th, and 14th days after trauma. **(B)** The rate of wound-closure on different days in wounds receiving different treatments. **(C)** On the 14th day after trauma, the wounds were treated with PBS or EPC exosomes of different concentrations and then stained with H&E (Zhang J. et al., 2016). **(D)** Representative photographs showing the effect of conditioned medium from days 0 to 6 on the transwell migration and tubule formation of HMEC-1 (Tao et al., 2017). **(E, F)** Control cells and 200 μg/ml wheat exosome (WE)–treated cells were incubated (37°C, 5% CO_2_) in DMEM medium supplemented with 10% FBS. Average number of branches formed by both control and treated cells (Sahin et al., 2019).

Exosomes accelerate tissue remodeling by activating endothelial cells and fibroblasts, promoting pro-angiogenesis and initiating extracellular matrix deposition (Zhang J. et al., 2015; Shabbir et al., 2015; Zhao et al., 2018; Ma et al., 2019; Xu et al., 2020). In mouse wound experiments, Zhang J. et al. (2016) injected exosomes derived from endothelial progenitor cells (EPCs) into a mouse abdomen and found that the EPCs-Exos can enhance human microvessels by activating the ERK1/2 signaling pathway. The ability of endothelial cells to proliferate, migrate and form tubes, thereby improving the rate of skin wound healing in diabetic rats, enhancing neovascularization, epidermal and collagen tissue regeneration, and then accelerating tissue remodeling (Figures 4A–C). It can be seen that synovial MSCs-EXOs can function in promoting the formation of blood vessels by activating protein kinase B (AKT) and extracellular signal-regulated kinase (ERK) pathways. Endothelial cells proliferate and promote fibroblast migration to the wound site and capillary angiogenesis through HSP70 and HSP90, accelerating tissue remodeling (Tao et al., 2017) (Figure 4D).

Additionally, it has been discovered that wheat-derived nano-vesicles can encourage human endothelial cell proliferation and migration and to improve the synthesis of endothelial cell tubular structures. Moreover, increased expression of Collagen 1 contributes to tissue remodeling (Sahin et al., 2019) (Figures 4E, F). The study of wheat-derived nano-vesicles provides a new solution for wound repair and lays a foundation for future research on wound repair mechanisms and the development of wound repair-related drugs.

#### 3.1.4 Inhibition of scarring

**FIGURE 5:**
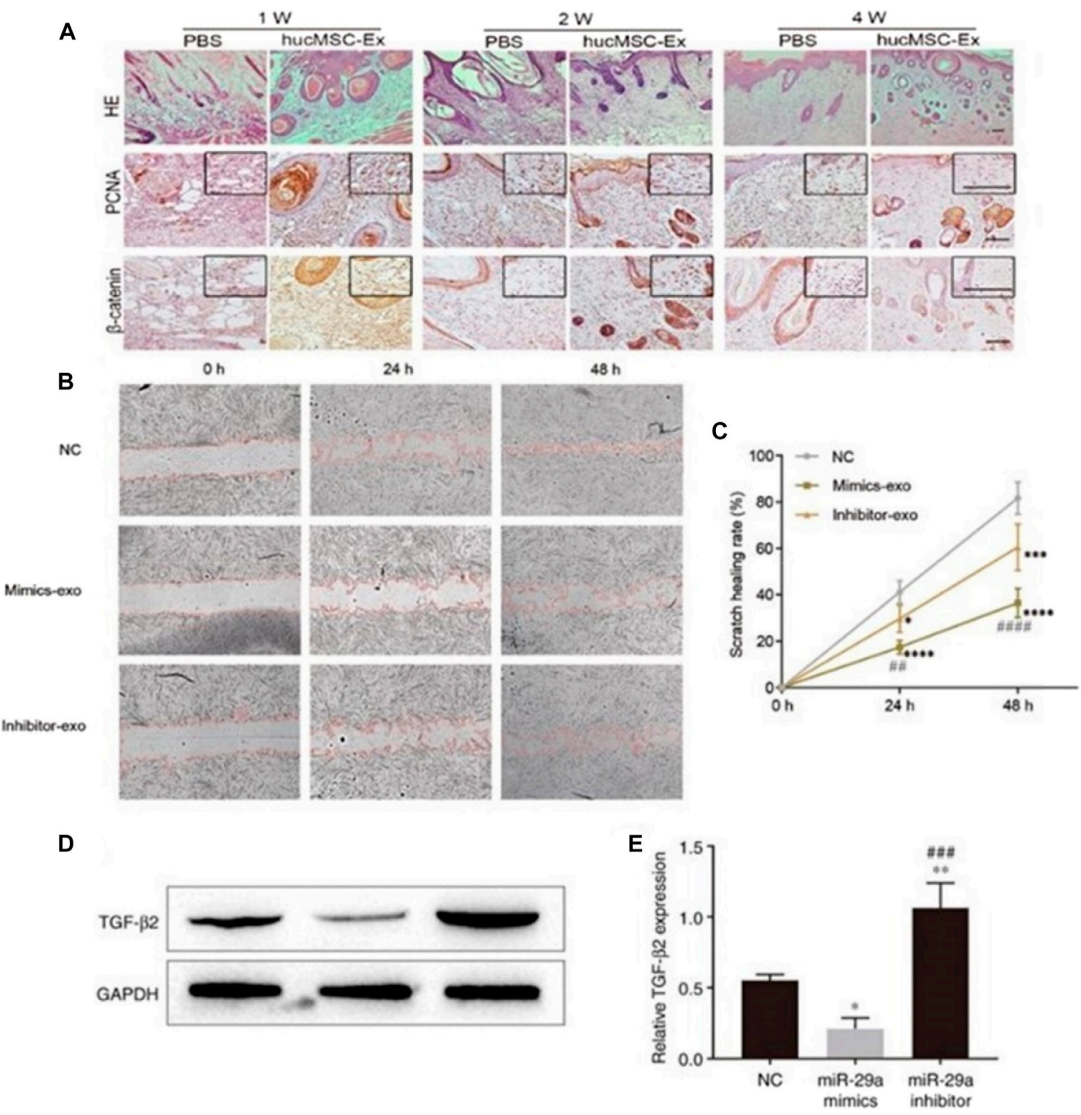
Exosomes inhibits the formation of scar by inhibiting the excessive proliferation of tissues. **(A)** Representative images of immunohistochemical staining of PCNA and β-catenin in each group. Scale bar = 100 μm (Zhang B. et al., 2016). **(B, C)** Effect of miR-29a-modified hADSCs-exo on the scratch healing of HSFBs. **(D, E)** Effect of miR-29a overexpression and knockdown on the expression level of TGF-β2 in HSFBs (Yuan et al., 2021).

Exosomes inhibit scarring by inhibiting tissue hyperproliferation. The study found that human mesenchymal stem cell-derived exosomes (hucMSC-Exos) promoted wound repair by activating the Hippo signaling pathway in a rat skin burn model, inhibiting excessive tissue proliferation and scar formation (Zhang B. et al., 2016) (Figure 5A). HucMSC-Exos also inhibits scarring by inhibiting the activation of TGF-β/SMAD2 pathway in fibroblasts by transporting miR-29a (Chen et al., 2019; Yuan et al., 2021) (Figures 5B–E). All of the above proves that exosomes are important medium for information transfer between cells and cytokines, which can be used as a new hope for cell-free therapy for non-invasive wound repair.

### 3.2 The role and mechanism of exosomes in anti-aging

The essence of human aging involves the aging of cells. Cell aging is due to the accumulation of damage that induces the activation of cell cycle inhibition pathways. Cells permanently exit the cell proliferation cycle, and cellular aging has permanent cell cycle arrest, reduction of NAD^+^, apoptosis resistance, aging-associated inflammatory cytokine secretion, and altered metabolic and epigenetic signatures (Kirkland and Tchkonia, 2017). Exosomes often contain biologically active proteins and genetic information, all of which play an important role in delaying cell aging (Liao et al., 2021), such as inhibiting the synthesis of related aging factor-β-galactosidase (SA-β-Gal) (Kim et al., 2021), and inhibiting skin photoaging (Gao et al., 2021).

#### 3.2.1 Exosomes delay cell senescence

**FIGURE 6:**
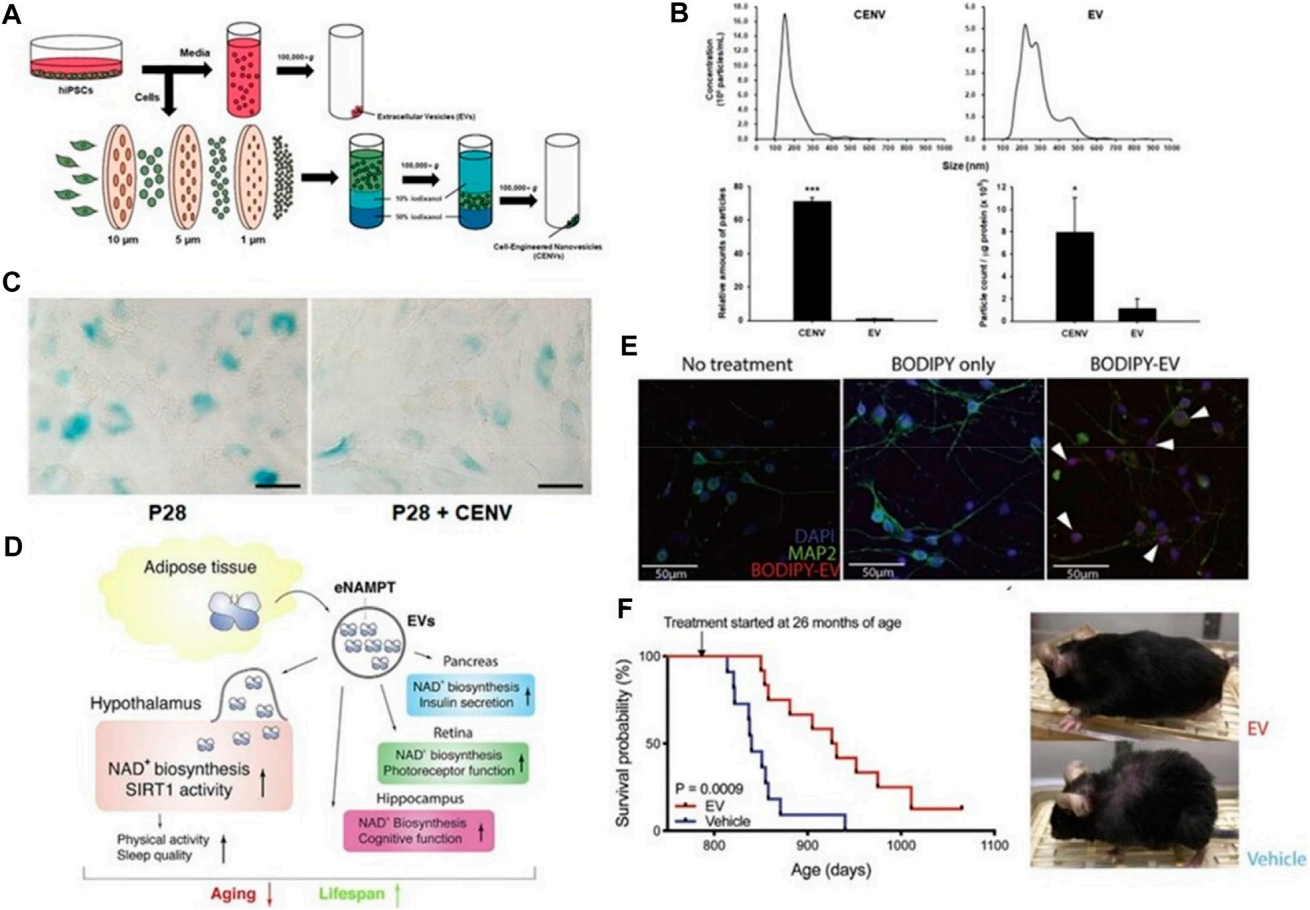
Exosomes delay cell senescence. **(A)** Schematic of the preparation of extracellular vesicles (EVs), and cell-engineered nanovesicles (CENVs) from human induced pluripotent stem cells (iPSCs). **(B, C)** Comparison of human iPSC-derived CENV and EV (Lee H. et al., 2020). **(D)** Mechanism Diagram of eNAMPT Containing Extracellular Vesicles Delaying Cell Aging. **(E)** Fluorescent images of primary hypothalamic neurons following the incubation with BODIPY-labeled EVs. **(F)** Kaplan-Meier curves and representative images of aged female mice injected with vehicle or EVs isolated from 4- to 12-month-old mice (*n* = 11–12). The mouse images were taken after 3 months of treatment (Yoshida et al., 2019).

Exosomes can delay cell senescence by inhibiting the synthesis of related senescence factor-β-galactosidase (SA-β-Gal) (Kim et al., 2021) and promoting the synthesis of nicotinamide adenine dinucleotide (NAD^+^) (McReynolds et al., 2021). Studies have shown that human-induced potent stem cell-derived exosomes (iPSC-EXO) can delay fibroblast senescence by inhibiting the synthesis of senescence-related factor-β-galactosidase (SA-β-Gal). Lee et al. (2020a) fabricated cell-engineered nanovesicles (CENVs) by continuously extruding iPSCs through membrane filters. They discovered that IPSC-CENVs had characteristics with IPSC-EXOs, and iPSC-CNEVs greatly diminished fibroblasts (HDFs), senescence-associated-galactosidase (SA-Gal), expression of p53 and p21, and SA-Gal activity, thus delaying cellular senescence (Figures 6A–C). These results suggest that the iPSC-CENVs can serve as an excellent surrogate for iPSC-EXO, and as well as a source of drugs for treating skin aging.

In addition, multiple studies have confirmed that NAD^+^ is a major player in cellular aging. Studies have shown that NAD^+^ levels in the circulatory system are decreased significantly with age in mice and humans during aging (Clement et al., 2019; McReynolds et al., 2020). Aging and muscle contraction enhance NAD^+^ utilization, and under normal physiological conditions, NAD^+^ depletion occurs primarily through salvage pathways catalyzed by nicotinamide phosphoribosyl transferase (NAMPT). Studies by Yoshida et al. (2019) have shown that blood transfusion into neonatal mice can prolong the lifespan and improve the health status of aging mice, mainly because NAMPT carried in blood exosomes increases the biosynthesis of NAD^+^, thereby prolonging the lifespan of mice (Figures 6D–F). These results indicate that NAMPT carried by blood exosomes has the effect of delaying cell senescence, laying a foundation for the development of later anti-aging drugs.

#### 3.2.2 Exosomes inhibit skin photoaging

**FIGURE 7:**
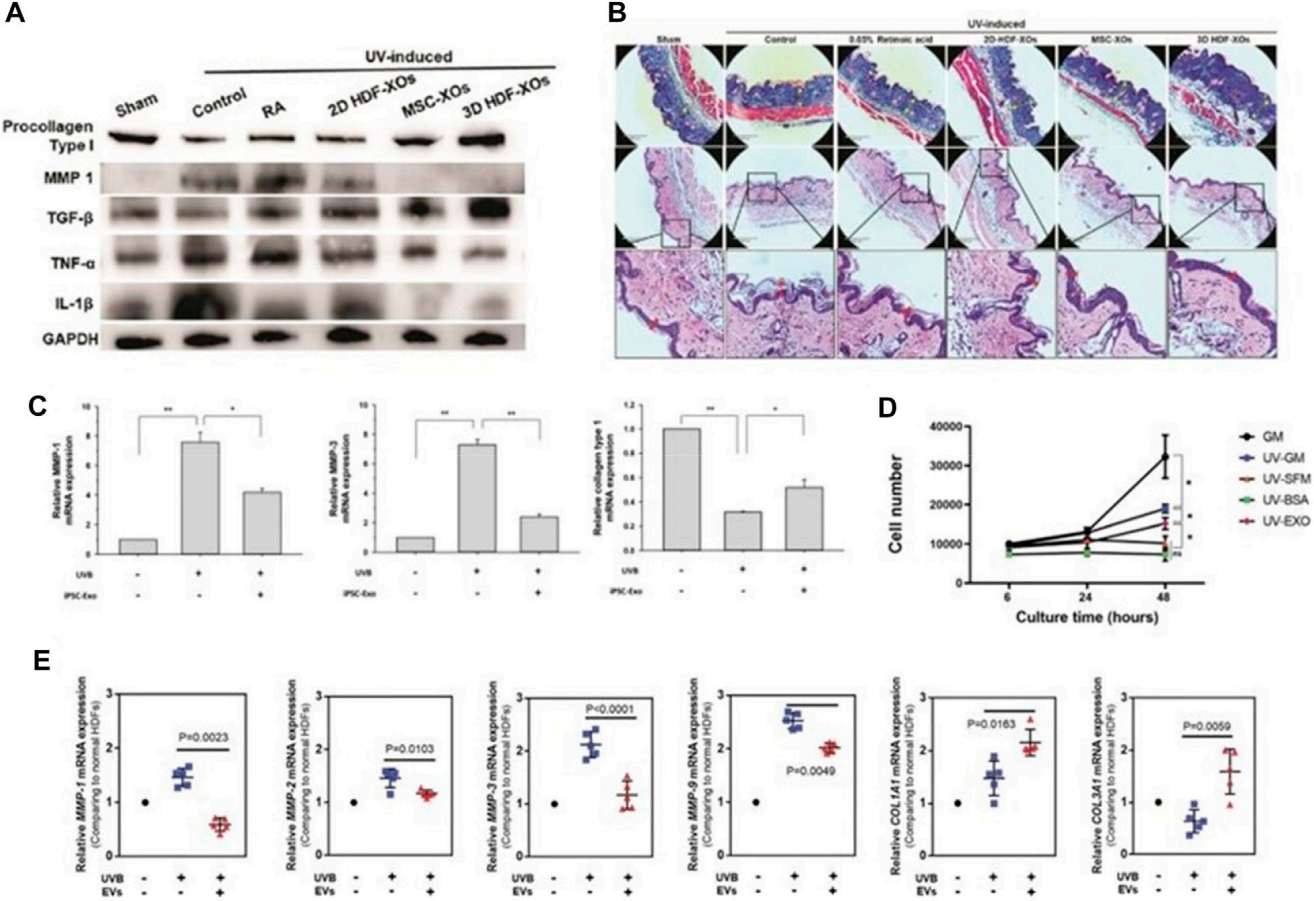
The mechanism for exosome regulation of MMPs. **(A)** Western blot of dorsal skin of different groups. **(B)** Masson’s Trichrome staining and Corresponding H & E staining. scale bar: 290 μm (Hu et al., 2019). **(C)** The mRNA expression levels of MMP-1, MMP-3 and collagen type I were quantified by quantitative real-time RT-PCR (Oh et al., 2018). **(D, E)** The proliferation of HDFs in different groups was measured by CCK-8 method. Gene expression in HDFs with or without HASC-derived EVs treatment after UV irradiation (Choi et al., 2019).

Skin photoaging is the most direct manifestation of human aging, mainly due to the abnormal up-regulation of matrix metalloproteinases (MMPs) after UV exposure, resulting in obvious skin wrinkles (Mazini et al., 2021). The researchers used a needle-free syringe to inject human dermal fibroblasts (HDFs) exosomes cultured in three-dimensional spheroids (3D-HDF-XOs) and monolayers (2D-HDF-XOs) *in vitro* and in a nude mouse model for photoaging, respectively. They found that the 3D-HDF-XOs cultured exosomes caused a significant decrease in MMP-1 expression and increased type I procollagen expression, mainly by down-regulating tumor necrosis factor-α (TNF-α) and up-regulating transforming growth factor-β (TGF-β) (Figure 7A). These findings imply that exosomes produced from 3D grown HDF spheroids have anti-aging capabilities and can also be used to treat and prevent the aging process of skin (Hu et al., 2019) (Figure 7B). In addition, human induced pluripotent stem cell exosomes (iPSCs-Exo) can inhibit UVB radiation-induced HDFs damage and matrix metalloproteinase 1/3 (MMP-1/3) overexpression. Increased expression level of collagen type I in photoaged HDFs was investigated (Oh et al., 2018) (Figure 7C). Exosomes (EXO) from human adipose-derived stem cells (HASCs) were also studied for their impact on photodamaged human dermal fibroblasts (HDFs), and it was discovered that adipose-derived mesenchymal stem cell exosomes (ADSCs-Exo) significantly inhibited UV-irradiation-induced overexpression of MMP-1, 2, 3, and 9, and enhanced I (from 119.6% ± 35.8% to 262.6% ± 54.1%), type III (from 61.2% ± 4.5% to 80.8% ± 2.7%) collagen and elastin expression (Choi et al., 2019) (Figures 7D, E).

These above results revealed that exosomes have the potential to be combined with cosmetics or drugs. However, the transformational application of exosome anti-aging effect is still in its infancy, and there is no clear clinical report, but its huge application potential deserves attention.

### 3.3 The role and mechanism for exosomes in hyperpigmentation

Asian women have long had a penchant for inhibiting hyperpigmentation (Hakozaki et al., 2002; Boo, 2022). Since skin color is mainly determined by the content and distribution of skin pigments, melanin is the most important determinant. Therefore, inhibiting melanin formation is one of the main ways to reduce skin pigmentation (Hwang and Hong, 2017). Human skin, mucous membranes, the retina, the pia mater, the gallbladder and ovary are all rich in melanin. Exosomes may have the effect of reducing hyperpigmentation by inhibiting the production of melanin (Lo Cicero et al., 2015).

**FIGURE 8:**
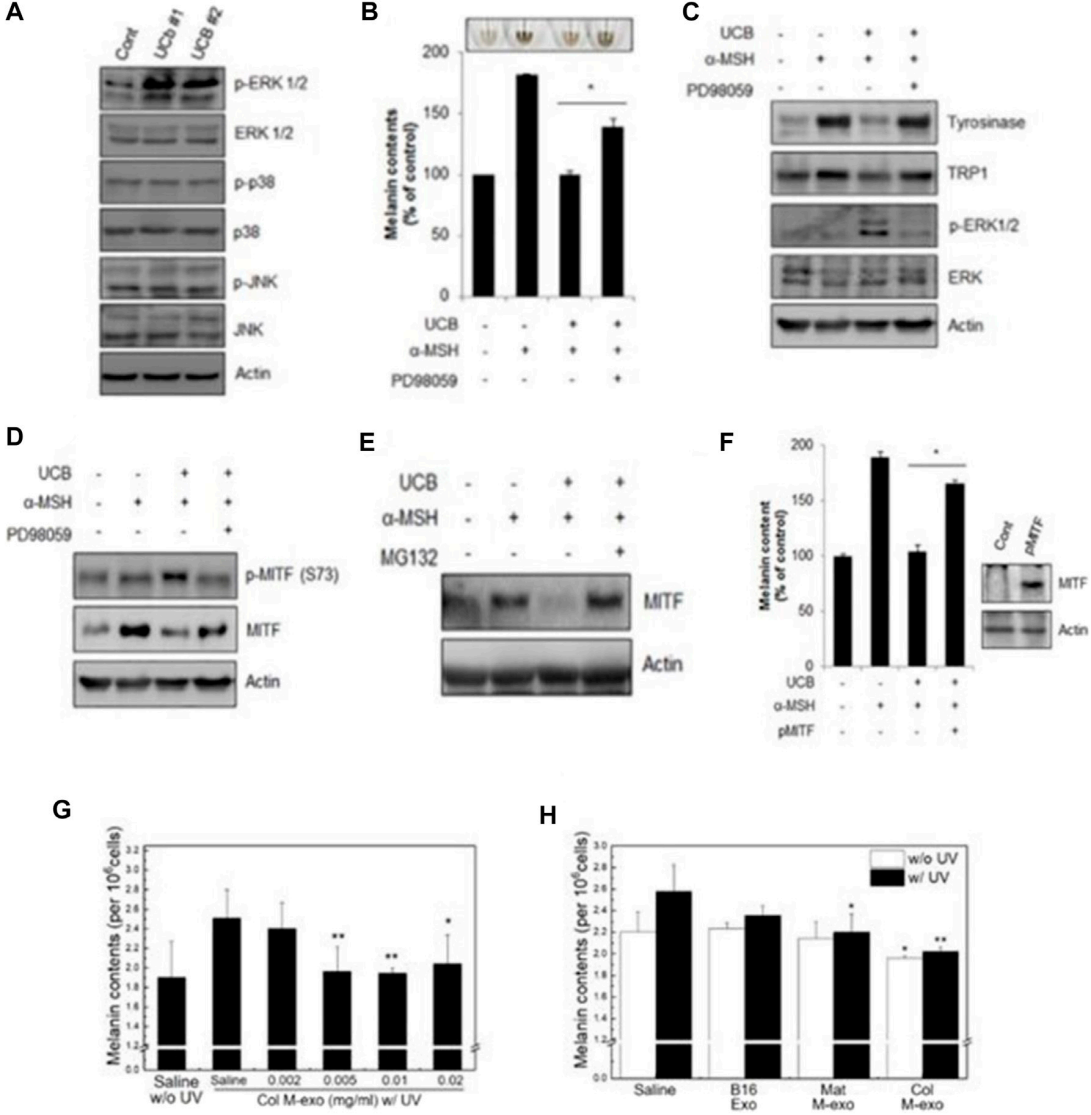
Exosomes inhibit melanin production. **(A–C)** Western blot was used to analyze the expression of related proteins. Melanin content in cells under different conditions.Western blot analysis of protein expression levels of tyrosinase, TRP1, p-ERK1/2 and ERK1/2 in cells. **(D–F)** Western blot analysis was used to detect the level of MITF under different conditions (Kim et al., 2015). **(G, H)** Melanin content of UV irradiated cells under different conditions (Han et al., 2022).

Studies have shown that miR-181a-5p and miR-199a in human amniotic stem cell-derived exosomes promote melanosome degradation in skin hyperpigmentation by inhibiting melanogenesis by reducing MITF expression, respectively. Exosomes derived from human umbilical cord mesenchymal stem cells are activated *via* the ERK pathway (Figures 8A–C), which significantly inhibits melanin synthesis during the degradation of MITF gene (Kim et al., 2015) (Figures 8D–F). In addition, miR-2478 in milk exosomes directly targets rap1a *via* the Akt-GSK3β pathway as a regulator of melanin production, reducing melanin content in melanocytes and thereby inhibiting the formation of melanin (Bae and Kim, 2021; Han et al., 2022) (Figures 8G, H). These results indicate that the exosomes can be used as an effective cosmetic ingredient to inhibit hyperpigmentation, and have great application potential in regulating skin pigment. The huge application potential of exosomes is expected to boost for the commercialization of exosomes based cosmeceutical products in the future.

### 3.4 The role and mechanism of exosomes in inhibiting hair loss

Hair loss is affected by various internal and external factors such as genetics, endocrine function (thyroid organ disease, changes in sex hormone levels), immune system diseases, malnutrition, drugs, mental state and natural aging. The above factors can affect the hair cycle (HC), reducing the activity and repair ability of hair follicle stem cells (Gentile and Garcovich, 2019). Dermal papilla cells (DPCs) are located in the hair bulb at the bottom of the hair follicle, surrounded by the dermal sheath and hair matrix cells, and are considered a unique type of mesenchymal stem cells. DPCs serve as signaling centers in the hair follicle (HF) and play an important role in regulating hair growth, formation, and circulation. Exosomes can inhibit hair loss by inducing HF regeneration by promoting the proliferation of DPCs (Kost et al., 2022).

**FIGURE 9:**
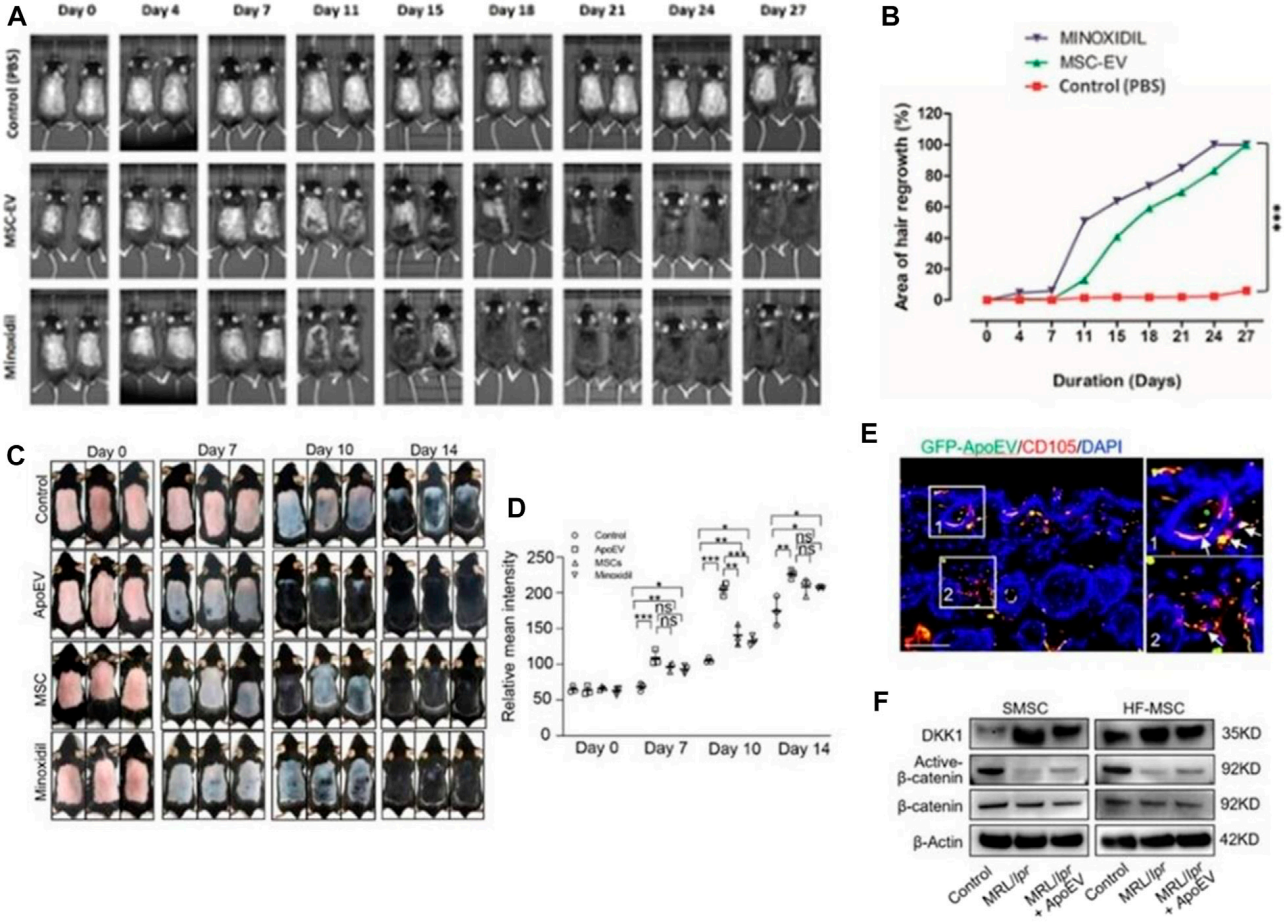
Hair regeneration induced by exosomes. **(A, B)** Take skin photos on days 0, 4, 11, 15, 18, 21, 24, and 28. Then, the hair regeneration area quantized by imageJ software is expressed in percentage (Rajendran et al., 2017). **(C, D)** Representative photos of mice showing skin color darkness and hair regrowth at 0, 7, 10 and 14 days post-injection. The level of pigmentation was quantified by the intensity of the darkness of the back skin in the same area. **(E)** Immunofluore-scent images show that GFP-ApoEV (green) were engulfed by skin MSCs (SMSCs) (1 and 2), as indicated by co-staining with CD105 at 7 days post-injection. **(F)** Western blotting shows Wnt/β-catenin signaling is activated and DKK1 expression is decreased in SMSCs and hair follicle MSCs (HF-MSCs) from MRL/lpr mice after apoEVs injection (Ma et al., 2023).

Studies have shown that the DPCs-Exos affect the hair follicle signaling pathway through the miRNAs it carries. By acting on key pathway genes such as β-catenin, Wnt, BMP2, BMP4, and downregulating related hair follicle inhibitory signaling proteins, the DPCs-Exos promotes hair follicle stem cell proliferation, hair follicle regeneration, and hair follicle formation. The telogen phase transitions to the growth phase (Zhou et al., 2018; Zhang et al., 2022). ADSC-Exos significantly promoted the proliferation and migration of DPCs by regulating miR-22, Wnt/β-catenin signaling pathway, and TNF-α signaling pathway, and promoting the expression of ALP, versican, and α-SMA proteins while maintaining their hair-inducibility, finally having a positive effect on hair follicle regeneration (Nilforoushzadeh et al., 2020). Human mesenchymal stem cell-derived exosomes activate the hair-inducibility of DPCs by stimulating Akt phosphorylation, and increasing Bcl-2 in the dermal papilla, thereby regulating the proliferation of DPCs and transforming hair follicles from dormant to anagen phase (Rajendran et al., 2017; Taghiabadi et al., 2020) (Figures 9A, B).

Recently, Kou et al. proposed for the first time that apoEVs play an important role in maintaining stem cell homeostasis *in vivo*. They found that exogenous apoEVs are metabolized through skin and hair follicles in a wave-like manner. At the same time, exogenous apoEVs can activate skin and hair follicle mesenchymal stem cells through the Wnt/β-catenin pathway to promote hair regeneration (Ma et al., 2023) (Figures 9C–F). All of the above implies that the exosomes may be a promising cell-free therapeutic strategy for immune-mediated hair loss (Li Y. et al., 2022). However, exosome-based treatments for hair loss are still in their infancy, and more robust clinical studies are needed to better evaluate their mechanisms of action, efficacy, safety, benefits, and limitations (Egger et al., 2020).

### 3.5 Clinical trials

At present, in the aspect of medical aesthetics, only 3 studies are based on the clinical trial registration related to wound healing of exosomes (17 November 2022) (Li et al., 2021a). The clinical trials of exosomes in anti-aging, inhibition of hyperpigmentation and hair loss have not yet been reported. Previous studies have found that exosomes from serum can accelerate the healing of skin wounds in BALB/c mice (Li et al., 2021b; Lee et al., 2021). Therefore, a trial is currently under way, in which autologous exosomes from plasma will be applied to the ulcer of the participants every day for 28 days. Then evaluate the healing of the skin wound according to the length, width and depth of the wound (ClinicalTrials.gov: NCT02565264). In addition, there is also a single arm pilot study. All patients were recruited on an outpatient basis. Debridement and photography were performed on the patient’s wound surface, and the wound area was measured. Then provide fat tissue exocrine dressing for patients in the intervention group. Exosomes were mixed with sterile hydrogel and applied directly to the wound surface, and the wound was covered with inert protective dressing. Patients who meet the inclusion and exclusion criteria at the end of the induction period will receive treatment twice a week for 4 weeks or until recovery (ClinicalTrials.gov: NCT05475418). To conduct a pilot clinical trial in diabetes patients with chronic skin ulcer (CCU), and evaluate the effect of the exosomes of mesenchymal stem cells (MSC) on the healing and regeneration ability of personalized nutritional supplementation (ClinicalTrials.gov: NCT05243368). Furthermore, there are currently five registered clinical trials based on plant exosome-like nanovesicles, including those from grape, lemon, aloe and ginger (ClinicalTrials.gov: NCT01668849; NCT04698447; NCT03493984; NCT01294072; NCT04879810), which are respectively studies on reducing the incidence rate of oral mucositis, cardiovascular risk factors, polycystic ovary syndrome, colon cancer and inflammatory bowel disease. Although there is no clinical trial related to medical aesthetics for these plant exosome-like nanovesicles, it has been reported that the plant exosome-like nanovesicles of grapefruit (Savcı et al., 2021), lemon (Manconi et al., 2016) and aloe (Zeng et al., 2021; Kim and Park, 2022) have potential application value in wound healing and anti-aging, and are expected to conduct clinical trials in the future (Leggio et al., 2020; Dad et al., 2021).

## 4 Administration of exosomes in medical aesthetics

At present, exosomes are mainly used for medical aesthetics research through topical application and local injection (Yari et al., 2022). Topical application (Li L. et al., 2020) can be directly applied locally to the skin or by means of microneedles (Zhang K. et al., 2020) or transdermal drug delivery (Shang et al., 2022) to increase the transdermal effect of exosomes. Local injection works mainly through subcutaneous injection (Cao et al., 2021). At present, there is no unified standard to judge which exosomes administration mode has the most obvious effect. The effect of exosomes from different sources may vary depending on the mode of administration. Therefore, which management mode to adopt is still a question and the focus of our discussion.

### 4.1 Topical application

Topical application can be applied to different areas according to different effects, and the dosage can be freely controlled. Xu et al. (2020) constructed a mouse skin injury model and found that both exosomes and miRNA-221-3p can promote wound healing in normal and diabetic mice by smearing endothelial progenitor cell exosomes or miRNA-221-3p. However, transdermal drug delivery can directly deliver active ingredients under the epidermis, opening the stratum corneum barrier, greatly improving the skin’s absorption rate of active substances, and reducing the loss of active ingredients. A detachable microneedle device-mediated drug delivery system for extended distribution of hair follicle stem cell activators was designed by Yang et al. (2017). The combination of this microneedle technology with small molecule medication UK5099 and exosomes produced from mesenchymal stem cells (MSCs) increased treatment efficacy at a lower dose and encouraged pigmentation and hair regrowth within six days through two rounds of treatment, according to their findings. In addition, this microneedle-based transdermal delivery technique demonstrated greater efficacy compared to topical UK5099 distribution and subcutaneous exosome injection. Additionally, human amniotic mesenchymal stem cells (hAMSCs) exosomes and hair nanoparticles were coupled with soluble microneedle patches (MNs) (HNP). They discovered that HNP-modified microneedle patches (HMNs) can efficiently permeate the stratum corneum and help deliver hAMSC exosomes into the dermis to stimulate proliferation of hair follicle stem cells and promote hair regeneration by causing the hair follicle to transition from telogen to anagen (Hong et al., 2021). Compared to direct administration, these combination transdermal treatments are more effective and safer (Shang et al., 2022). Some regenerative medicine companies, such as BENEV and Kimera Labs, have developed health care products involving exosomes for skin damage, anti-aging, pigment regulation and inhibition of hair loss. These products have been approved by the US Food and Drug Administration (FDA) (Peña-Juárez et al., 2022).

### 4.2 Local injection

Local injection has advantages such as less adverse reactions and simple operation. Here we mainly discuss subcutaneous injection. Han et al. (2021) *in vitro* studies on mice revealed that subcutaneous injection of DF-Exo markedly expedited the healing of diabetic skin lesions by boosting re-epithelialization, collagen deposition, skin cell proliferation, and angiogenesis. In addition, according to Shin et al., subcutaneous injection of adipose-derived mesenchymal stem cell exosomes significantly decreased trans epidermal water loss, increased stratum corneum (SC) hydration, and significantly decreased the expression of inflammatory cytokines like IL-4, etc., promoting wound healing (Shin et al., 2020). In addition, exosomes are often incorporated into hydrogels to function. Some researchers assembled MSC-derived exosomes in chitosan/silk hydrogels (Shiekh et al., 2020) and self-healing antibacterial polypeptide hydrogels (Wang C. et al., 2019), and found that exosome-assembled hydrogels can promote wound healing in diabetic patients and can also be placed directly in or near the target area, the dose of exosomes is more concentrated and more targeted (Riau et al., 2019). It has been shown to have the ability to rapidly heal wounds and is more effective than single exosome therapy, and reduces scarring (Wang C. et al., 2019; Wang M. et al., 2019). These results suggest that the exosomes can potentially be used as therapeutic agents to repair skin damage. However, there are still many problems, such as standardization of research methods, clarification of the mechanism of action, and validity of application (Mehryab et al., 2020). There are a few clinical studies on exosomes in medical aesthetics. At present, it is still in the primary stage of research (Jing et al., 2018; Hade et al., 2021) and has not been approved by the US Food and Drug Administration (FDA).

The above results indicate that exosomes can play a role in the field of medical aesthetics through different delivery methods. However, in terms of medical aesthetics, we recommend transdermal drug delivery. Compared to the direct application of exosomes, transdermal drug delivery can penetrate the cuticle, greatly improve the skin’s absorption rate of effective substances, and reduce the effective loss of ingredients. Compared to the injection of exosomes, transdermal drug delivery is more convenient, has fewer side effects, and is safer. It has huge application potential in the future medical beauty industry (Yang et al., 2021).

## 5 Exosome engineering production and preservation

The application of exosomes in medical aesthetics is inseparable from their large-scale production and preservation. The unique heterogeneity and other characteristics of exosomes hinder their engineering production. Secondly, different separation, purification and preservation methods have a greater impact on the yield and active components of exosomes. Therefore, it is crucial to adopt appropriate engineered production, separation and purification techniques and preservation methods of exosomes. Next, we specifically discuss the engineering production, separation, purification technology, and preservation methods of exosomes in recent years, laying the foundation for their application in medical aesthetics in the later period.

### 5.1 Exosome engineering production technology

At present, many companies and researchers are working hard to develop engineered exosomes for medical aesthetics (Gimona et al., 2017). To improve cell-to-cell communication, Cha et al. (2018) established the usage of a polyethylene glycol (PEG) hydrogel for cell aggregation and MSC spheroid formation. In addition, they employed a programmable dynamic culture method in the planned microwells, which increased EV production 100 times more than 2D cell culture. Cao et al. (2020) utilized a three-dimensional (3D) culture system of hollow fiber bioreactors to produce MSC-exos and evaluated the therapeutic effect of 3D-exosomes (3D-exos) on acute kidney injury (AKI). They discovered that compared to 2D culture, 3D culture did not significantly change the surface indicators of MSCs or their morphology, size, or exosome labelling. Tubular epithelial cells (TECs) absorbed the 3D-exos more effectively, leading to better anti-inflammatory efficacy and increased TEC viability *in vitro*. The total exosome yield was also raised by 19.4 times in the 3D culture system, and the hollow fiber technology provides benefits, including exosome concentration, immunity to serum exosome contamination, and a reduction in other contaminants. Based on the advantages of 3D culture technology, Enze Kangtai Company has successfully built an engineered exosome research and transformation platform with independent intellectual property rights - ModiExo^TM^. Based on mature site-directed insertion technology and backbone plasmids, the ModiExo™ platform can rapidly construct exosome scaffold protein-engineered cell lines and combine with 3D microcarrier culture technology to efficiently and stably mass-produce projects targeting specific organs or loading specific proteins/RNAs exosomes. The establishment of this platform has promoted the rapid development of exosome clinical transformation.

In addition, by using MYC gene to genetically modify human embryonic stem cell mesenchymal stem cells, we have created immortal cell lines that can permanently produce exosomes without batch renewal. However, this approach has not been well received by other researchers, and a biological function investigation alone cannot vouch that the exosomes’ characteristics are unaffected by cell transformation. Therefore, additional research is necessary to ascertain the potential alterations in exosome content (Chen et al., 2011). Therefore, the generation of exosomes by manipulating cellular sources or conditions requires further characterization of their composition and provides ideas for the development of new methods for exosomes in medical aesthetic applications.

However, the engineering application of exosomes still faces many challenges in medical aesthetics. At each step of large-scale exosomes production should be according to good manufacturing practice protocol (GMP). Further exploration is still needed in this field because systems based on GMP protocols and adequate quality control, and quality certification processes are still essential (Lener et al., 2015).

### 5.2 Engineering isolation and purification of exosomes

The putative use of exosomes in medical cosmetics has been continuously explored as exosome research has progressed. Exosome enrichment and reproducible isolation will make it easier to establish their biological activities (Zhang Y. et al., 2020). Conversely, exosomes collection and purification are complicated because exosomes from diverse sources fluctuate in size, composition, and function (Kalluri and LeBleu, 2020). Consequently, one of the current biggest issues is how to efficiently enrich exosomes, which is vital for the engineering production of exosomes. For varied uses and goals, different separation techniques are frequently used. At present, ultracentrifugation technology is one of the frequently employed exosome separation techniques in medical cosmetic applications (Livshits et al., 2015), polymer precipitation technology (Domenis et al., 2017), immunoaffinity capture technology (He et al., 2017) and asymmetric flow field-flow fractionation technology (Yang et al., 2017; Willms et al., 2018). However, the equipment required for ultracentrifugation technology is expensive and complex to operate. The polymer precipitation technology may be contaminated with other proteins and the purity of exosomes is low. The reagents required for immunoaffinity capture technology are expensive, and the isolated exosomes may lose activity. Asymmetric flow field-flow fractionation technology requires large sample size and low yield, which is easily limited in medical cosmetic applications (Yang X. X. et al., 2019; Alzhrani et al., 2021) (Table 4 for details)

**TABLE 4:** Isolation of exosomes.

Isolation technique	Isolation principle	Advantages	Disadvantages
Ultracentrifugation Techniques	Precipitate and isolate exosomes based on density and size	Separation of gold standard exosomes, and the most widely used	Time consumption, high cost, structural damage
Ultrafiltration technique	Filtration with different relative molecular mass	Low cost, high enrichment efficiency and exosomes activity are not affected	Low purity and non-specific bind-ing of exosomes and reduced recovery through exosomes and ultrafiltration membranes
Size-based isolation techniques	This technique mainly refers to ultrafiltration and size exclusion chromatography. Both are exclusively based on the differences in diameter between exosomes and other components	The structure and integrity of exosomes separated from SEC are usually not affected by shear force	It usually requires a long running time, which limits its application in processing a variety of biological samples
Polymer precipitation	Collect exosomes under centrifugation by reducing the solubility of exosomes, such as PEG	Relatively easy to operate with short analysis time and is suitable for processing large doses of samples	Expensive and other non-exosomal contaminants proteins and polymeric materials may co-precipitate, resulting in low purity exosomes
Immunoaffinity capture techniques	Based on specific binding of antibodies and ligands to separate desired substances from heterogeneous mixtures	This method is applicable to the isolation of exosomes with the same membrane protein expression, as well as the qualitative and quantitative determination of exosomes	The reagent cost is high. This method is not suitable for the mass separation of exosomes, and the separated exosomes may lose their activity
Microfluidics-derived chip isolation techniques	It is a microscale separation technique based on difference between biochemical and physical properties of exosomes, such as density, size and immunoaffinity	Fast sample processing, low cost and portability	Low yield, high sensitivity, only for diagnostic purposes, and a large amount of raw materials are needed to increase the output
Flow field-flow fractionation (FlFFF)	Particle size separation of sample components is realized by increasing the molecular weight or hydrodynamic diameter	Once the lipid target or marker is determined, you can use FlFFF ESI MS/MS for top-down lipid analysis to directly analyze the lipids in the exosomes without collecting the exosomes for lipid extraction	The lack of adequate sample validation has its limitations

Secondly, the research progress of exosomes in medical aesthetics is hindered to a certain extent by the heterogeneity of their application preparation process, and the purification efficiency of different separation methods is significantly different (Hettich et al., 2020). It is said that, size-based separation techniques, such as gravity size exclusion chromatography (SEC), are a faster purification method with higher purification efficiency and can obtain high-purity and intact exosomes (Lobb et al., 2015; Nordin et al., 2015; Mol et al., 2017), which are favored by many researchers. Size exclusion chromatography (SEC) was employed by Vaswani et al. (2017) to concentrate milk-derived exosomes from extracellular carriers (EVs) that could be enriched in about 30 min, and exosomes produced by SEC yield (3 × 10^12^ particles/ml) was higher that density gradient centrifugation (7 × 10^10^ particles/ml). However, there may be contamination of microvesicles in it.

**FIGURE 10:**
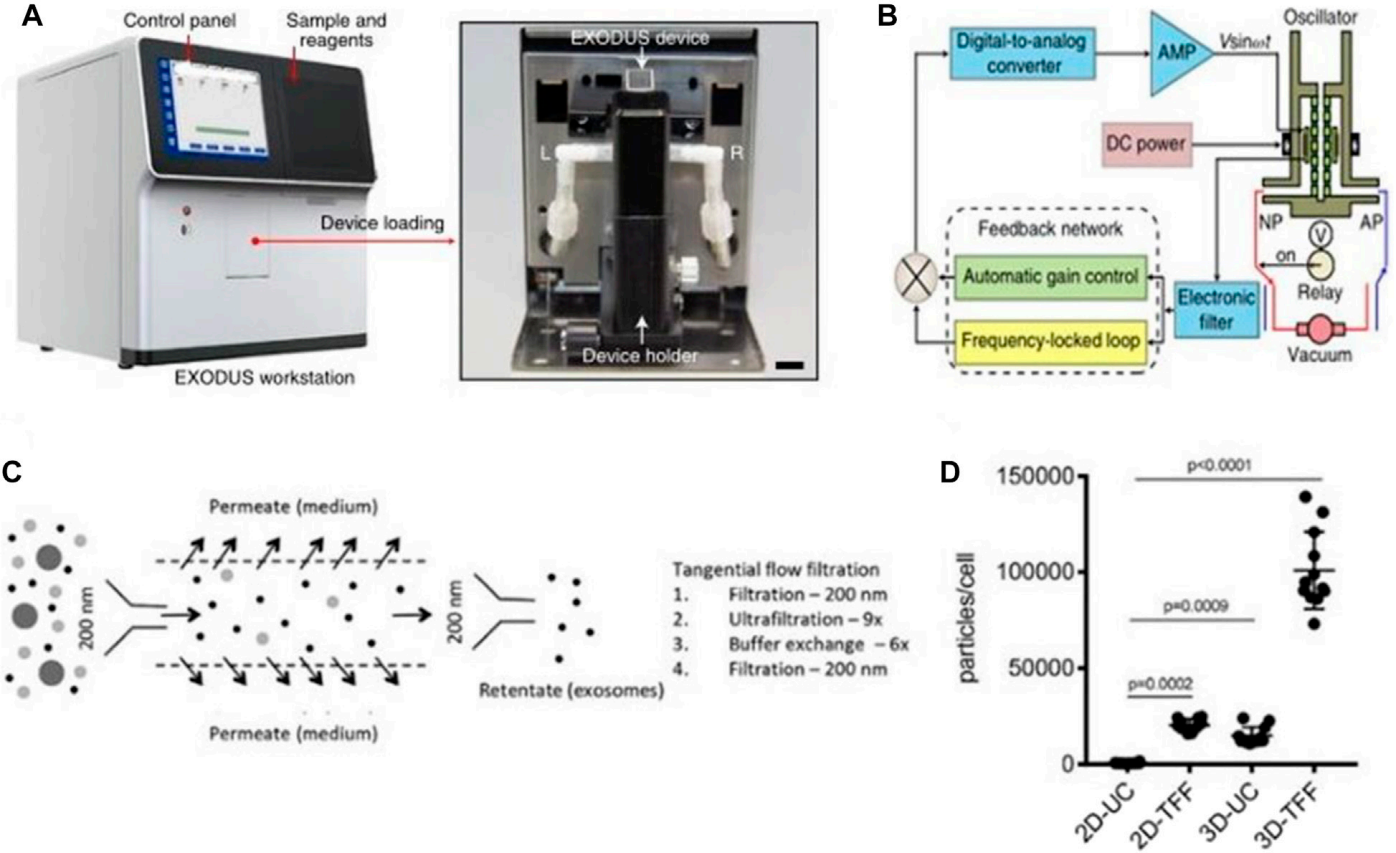
Exosomes separation device. **(A, B)** A photograph of the EXODUS station and Schematic diagram of the control module for the resonator (Chen et al., 2021). **(C, D)** Separation of exosomes by tangential flow filtration. Exosomes were enriched from the culture supernatant by tangential flow filtration with a 500 kDa cut-off filter cartridge (Haraszti et al., 2018).

Chen et al. (2021) invented an exosome detection method *via* ultra-fast separation system (EXODUS) (Figures 10A, B). EXODUS is based on the creation of negative pressure switching technology (NPO) and NPO-HO technology that simultaneously generates high and low-frequency vibration (HO) on nanomembranes and devices. Negative pressure oscillation and membrane vibration activated by double-coupled harmonic oscillators were used to ultra-effectively purify exosomes. This approach can quickly finish exosome separation in less than 10 min, compared to existing separation and purifying techniques. Exosomes isolated by EXODUS had the highest signal intensities from all chosen exosomal proteins and the lowest signal intensities from chosen protein contaminants, demonstrating the benefits of exosome isolation and purification. Concerning a variety of sample types, sample quantities, and exosome concentrations, the EXODUS system enables reproducible procedures, and reproducible results.

In addition, tangential flow filtration (TFF) is also considered to be an ideal method for engineering the isolation of exosomes (Reiner et al., 2017; Ha et al., 2020a; Ha et al., 2020b; Yi et al., 2020). The TFF system complies with Good Manufacturing Practice (GMP), which is a method for separating exosomes, concentrated proteins or viruses from a large number of cell culture media. (Potter et al., 2014; Haraszti et al., 2018) (Figures 10C, D). Lee et al. (2020c) used TFF technique to isolate exosomes from adipose tissue-derived adipose-derived mesenchymal stem cells on a large scale with positive effects on kidney injury. Several studies have proved that the yield and purity of TFF-isolated exosomes are comparable to methods based on size exclusion chromatography (Reiner et al., 2017; Busatto et al., 2018; Haraszti et al., 2018; Ha et al., 2020a; Ha et al., 2020b; Yi et al., 2020) and have been gradually applied in the field of medical aesthetics.

There is no absolute best separation method for exosomes, and their separation effect is closely related to downstream applications and scientific issues, but high recovery and high specificity are two recognized basic requirements. In addition, the complexity of the operating procedure, extraction cost, biological activity, and throughput still need to be considered. Due to the limitations of the separation principle, the current exosome separation and purification technology has bottlenecks such as contamination with other components and exosome aggregation. Therefore, selecting appropriate methods for exosome isolation from different sources according to their characteristics is crucial.

### 5.3 Preservation of exosomes

Exosome utilisation in medical aesthetics depends on a suitable storage environment. It is an essential step to keep the biological activity stable throughout preservation. Exosome function may be more affected by various storage conditions. It is crucial to maintain a specific storage state. Currently, cryopreservation, freeze-drying and spray-drying are the three main preservation procedures used.

#### 5.3.1 Cryopreservation

Cryopreservation is currently the storage technique that is most frequently employed (Mahdavinezhad et al., 2022). In order to preserve the functional stability of biological microparticles, a storage method known cryopreservation lowers the temperature below that needed for biochemical reactions. It is typically used at temperatures of 4°C, −20°C, −80°C, and −196°C (Saadeldin et al., 2020). But this way of preservation is prone to “frost burn”. The term “frozen injury” used here refers to ice crystal development inside biological particles and the imbalance of osmosis that occurs during freezing. In addition, repeated freezing and thawing changes the biological properties, content and marker composition of exosome surface molecules (Maroto et al., 2017). Therefore, an appropriate concentration of antifreeze is often selectively added to prolong shelf life. The most effective disaccharide antifreeze for exosomes is trehalose, which is mentioned as the best alternative (Nakanishi et al., 2020). Trehalose and other membrane stabilisers have been used to store labile proteins, vaccines, liposomes, and cryopreserve tissues and stem cells, thus it makes sense to utilise them to keep exosomes stable (Zhang X. G. et al., 2015; Bosch et al., 2016; Shinde et al., 2019). Budgude et al. added 25 mM trehalose to exosomes in PBS at −80°C and found that these cryopreserved exosomes could be taken up by hematopoietic stem cells as efficiently as freshly isolated exosomes and that trehalose was as effective forfreshly isolated exosomes (Budgude et al., 2021). The addition will not alter the form of the exosomes and can successfully prevent exosome aggregation during the freeze-drying process, boosting stability.

#### 5.3.2 Freeze drying

For heat-sensitive molecules such as proteins, peptides, vaccines, colloidal carriers, vesicles, and viruses, freeze-drying is currently thought to be the most dependable technique (Hansen et al., 2015; Muramatsu et al., 2022). In order to meet storage needs, a process known as freeze-drying pre-cools materials containing moisture to below freezing point, causes them to solidify. The ice is then directly sublimated in a vacuum and removed as water vapour Wang et al. (2022) have developed an inhaled, room temperature-stable virus-like particle (exosome) vaccine as a promising COVID-19 vaccine candidate. The new coronavirus vaccine consists of a recombinant new coronavirus receptor-binding domain (RBD) that binds to pulmonary exosomes, which can enhance the retention of RBD in the respiratory tract and lungs, and the new coronavirus vaccine can be lyophilized at room temperature for more than 3 months. However, the freezing and dehydration pressures generated during freeze-drying may cause the molecular structure of biomolecules to be destroyed. Therefore, it is also necessary to selectively add antifreeze, such as trehalose, to protect biological materials.

#### 5.3.3 Spray drying

Simple, quick, repeatable, and scalable drying methods include spray drying (Arpagaus et al., 2018). Compared to freeze-drying, spray drying is a continuous process, which does not require freezing steps, and can directly transform various liquids into solid particles with adjustable size, distribution, shape, porosity, density and chemical composition. Powders that have been sprayed-dried are of a high calibre, often have a reduced moisture content, and are more stable (Arpagaus et al., 2018; Kusuma et al., 2018). However, spray drying is currently mainly concentrated in food applications and capsule and tablet applications, and the preservation and transportation of exosomes have not yet been covered. If it can be applied to the field of medical aesthetics in the future, it will solve a major problem in the preservation and transportation of exosomes.

Overall, research is underway to determine the ideal storage conditions for exosomes in medical aesthetics. Exosomes should be kept at −80°C for long-term storage and at 4°C for short-term storage. For preparation and transportation of exosome-related drugs, a freeze-drying method is used, but cryoprotectants such as trehalose, albumin., need to be added during the freezing process to reduce the loss of extracellular vesicles during separation and preservation (Bosch et al., 2016).

## 6 Conclusion and future perceptive

This paper discusses the regulatory mechanism of exosomes in wound repair, anti-aging, inhibition of hyperpigmentation and hair loss, also summarizes the engineering production technology, administration methods, and preservation methods for exosomes in medical beauty, with further explanations of exosomes applications in the medical aesthetics industry.

As a new type of tissue engineering material, exosomes have been gradually applied in the medical aesthetics industry. However, the research on the function of exosomes is still in the early stage, and there is an urgent need to conduct comprehensive research on the functions of exosomes in absorption, distribution, and metabolism (Cheng et al., 2020). Second, the uncertainty about the accuracy and content of components in exosomes (Lee et al., 2012), such as miRNA, proteins, and lipids, their high cost for separation procedures, and low purity of exosomes have always been problems in their application in medical aesthetics (Golchin, 2021).

The widespread use of exosomes still faces several difficulties, which we must continue to investigate in the future from various angles. First, stem cell exosomes are the main exosomes used in medical aesthetics. However, more research is needed to determine the significant usefulness of a wide range of exosomes, including plant nanovesicles and microbial nanovesicles. The route and dose of administration of exosomes will also affect the biological distribution pattern (Wiklander et al., 2015). As a drug delivery carrier, how to achieve local administration suitable for home use and how to improve its efficacy is still a problem worth exploring. Secondly, the small automatic preparation device has a huge application market at home, and how to develop a small automatic exosome extraction device suitable for home use is also a direction worth exploring. At the same time, large-scale preparation technology combined with long-term effective preservation technology is also a direction worthy of research.

Exosomes still face many challenges in clinical practice. In clinical practice and medical aesthetics, allogeneic exosomes are often applied to individuals. Although allogeneic exosomes can induce T cell proinflammatory allogeneic immune response *in vitro* and *in vivo*, due to different donors, the drug formation ability of allogeneic exosomes needs further study. (Marino et al., 2016; Lu et al., 2019). How to improve the curative effect of exosomes is the key point worth studying. In addition, due to heterogeneity and donor age, sex, body mass index, drug use, race and other factors that affect the level of exosomes (Witwer et al., 2013). With the in-depth study of engineered exosomes, the selection of engineered cells for different indications, production standards and standardization of clinical trials are also an important direction.
